# When Imagination Feels Like Reality: A Case Study of False Memories and Maladaptive Daydreaming in Visual Impairment

**DOI:** 10.1155/2024/9391645

**Published:** 2024-04-09

**Authors:** Eli Somer

**Affiliations:** School of Social Work, University of Haifa, Haifa, Israel

## Abstract

**Background:**

When a person experiences maladaptive daydreaming (MD), they spend a prolonged period daydreaming with a strong sense of presence. The symptoms of MD are often excessive, interfere with functioning, and are linked to distress and comorbid mental disorders. In this paper, apparent false memory is described in the context of a woman with MD and visual impairment due to a progressive eye condition. Her vivid daydreams seemed indistinguishable from actual memories. *Case Report*. A 35-year-old woman with a lifelong MD reported three incidents of fabricating detailed false memories of events that her family confirmed never occurred: obtaining a new job, miscarrying twins, and hospitalization for COVID-19. She experienced anxiety and shame when the stories were disproven. The assessment confirmed MD, PTSD, OCD, and other disorders. Her verbal memory was below average, especially for longer narratives. Her misattributions of daydreams as real-life memories may relate to reliance on vivid mental images over deteriorating vision and source monitoring deficits.

**Conclusion:**

This first reported case of confabulations in an individual with MD and visual disability suggests daydreams could potentially be mistaken for actual events in some MD cases. While sensitive, more research is needed on the prevalence of false memories among individuals with MD. The default mode network, prefrontal cortex, and their connectivity may be implicated in generating vivid daydreams and misattributing them to actual episodic events. Understanding the relationship between sensory impairments, dissociation, and susceptibility to memory distortions could inform interventions to improve reality testing for some MD patients.

## 1. Introduction

This case report describes an instance of apparent false memory formation in Adah, a woman with maladaptive daydreaming (MD) and visual impairment due to a progressive eye condition. Her vivid daydreams seemed indistinguishable from actual memories. Through Adah's case, we aim to investigate the potential relationship between MD and the development of false memories.

Prolonged compulsive immersion in vivid fantasy featuring complex scenarios characterizes MD. A vivid, time-consuming habit can develop from immersive daydreaming (ID) due to its gratifying content [1, 2]. Gone awry, MD interferes with essential functioning such as study, work, and social or family life [3, 4]. MD is conceptualized as a dissociative absorption process [5]. Instead of temporarily distancing from the self by numbing perception, as in depersonalization/depersonalisation disorder, or employing an identity alteration, as in dissociative identity disorder, the MD process involves temporarily distancing from the self and its immediate experience by experimenting with alternate protagonists/identities. This detachment is achieved through a division of streams of consciousness, as demonstrated by “absorption and imaginative involvement” or “dissociative absorption” [5].

Somer et al. [6] showed evidence that the phenomenology of MD could be sensually quite similar to external reality. For example, some of their respondents said, “It feels like a normal conversation like me, and you are having”; “It is like a reality with colors, smells, and tastes. I can hear outside noises, but I can block them out” and, “I daydream with open eyes…but I can clearly see and hear my daydream as if it were real” (P. 571), because MD imagery is not only sensorily rich but also affect-laden. Having many properties of real-life experiences, it is only surprising that 98% of participants stated that they did not confuse fantasy and reality [7]. Still, the following case reported in this paper suggests that such misremembrance is possible in MD. This report enters into the long-standing controversy over the malleability of memory and susceptibility to false memories [8]. Despite the rarity of such confusion in MD patients, documenting an instance of misattributed fantasy as reality breaks new ground in the literature.

## 2. Case Report

Adah is a 35-year-old single woman who was adopted as an infant. She has a high school education and attended some college classes. Adah was recently bereaved by the loss of her beloved adopting mother to cancer. At age 7, she was diagnosed with retinitis pigmentosa, a genetic disorder causing progressive vision impairment, which has hindered her independence. Adah has a history of a dysfunctional, violent, long-term relationship with an abusive man. Since the COVID-19 pandemic, she has experienced further isolation due to social distancing restrictions and developed contamination fears and compulsive cleaning rituals. She is unemployed and financially dependent on social security benefits and her father's support. Adah's father initiated contact, expressing concern about her confabulating fantasies that seemed indistinguishable from reality, causing turmoil among family members.

Adah is a likable but avoidant woman who oversleeps and overeats. She has a lifelong habit of vivid daydreaming, often involving Disney characters, which provides an escape from her emotional and visual darkness. Despite acknowledging her tendency to “sugar-coat” her problems, Adah showed reluctance to explore her inappropriate fantasies or other sources of distress, such as PTSD, visual impairment, and social isolation. After recounting three fabricated memories that caused serious family turmoil, she was referred for psychological diagnosis and therapy.

### 2.1. Psychological Evaluation

In assessing Adah's condition, I administered the Wide Range Assessment of Memory and Learning (WRAML-2), selected subtests [9], the Minnesota Multiphasic Personality Inventory (MMPI-2) [10], the 16-item Maladaptive Daydreaming Scale (MDS-16) [11], and the Structured Clinical Interview for Maladaptive Daydreaming (SCIMD) [12]. The patient demonstrated below-average performance on the WRAML-2 Word Lists subtest, which measures rote verbal memory through memorization of word lists. She also showed below-average ability to remember phrases spoken to her accurately. Notably, when recalling word lists, Adah would sometimes introduce new words that she repeatedly believed were included on the list despite re-exposure to the actual list. She exhibited considerable difficulty with free recall of longer narratives, initially scoring far below average. However, Adah displayed somewhat better memory for narratives when either the presentation rate was slowed, and she repeated sections before the final recall or when recall cues were provided. Overall, the findings indicated significant challenges with verbal memory, particularly in the recall of more substantial amounts of information.

Adah's memory improved somewhat when presentation rates were slowed. She was allowed to repeat sections before final recall or when recall cues were provided. This pattern suggests her verbal memory deficits may be related to issues with attention and focus. Adah's experiences of dissociative absorption in fantasy could interfere with her ability to attend to and encode verbal information presented to her fully. This dissociative tendency may contribute to her memory confabulation, where she falsely believed certain words or details were part of the original information.

Adah completed the MMPI-2 in its entirety, demonstrating adequate effort and comprehension. However, validity scales revealed an unusual profile across multiple subscales. Her responses reflected atypical symptom reporting. While she presented as cooperative with no evidence of intentional minimization or defensiveness, the possibility of overreporting due to emotional needs cannot be excluded. Overall, Adah's clinical scale profile indicated primary elevations related to anxious and persecutory thinking, with additional elevations on scales measuring depression, post-traumatic stress, and obsessive–compulsive symptoms. Subscale analysis also suggested tendencies toward social isolation, alienation, and interpersonal distrust. In summary, she exhibited a range of internalizing symptoms centered around anxiety, social disconnection, and difficulty trusting others.

Adah exhibits a profile that suggests significant psychological and emotional difficulties, which may be related to her reported fabrication of events and memories. The MMPI-2 indicates the possibility of overreporting symptoms due to emotional needs rather than an accurate representation of her psychological state.

Adah's clinical scale profile showed a range of significant internalizing issues that are likely causing her substantial distress. The subscale analysis also suggested tendencies toward social isolation, alienation, and interpersonal distrust. These symptoms raise concerns about Adah's ability to form and maintain healthy relationships, which could further exacerbate her loneliness and need for acceptance. Given the MMPI-2 profile, the patient's reported tendency to fabricate events and memories involving successful employment and medical hospitalizations may represent a pathological coping mechanism. The elevated scales suggest Adah may be struggling with significant emotional and psychological challenges that she is attempting to manage through the creation of an alternative narrative.

The evidence-based cutoff score for suspected MD on the MDS-16 is 40 [8, 13]. Adah scored 68 on the test, indicating a high likelihood of having the condition. Subsequently, I interviewed Adah using the SCIMD [14], which confirmed that she was positive for MD, severe. Additionally, based on self-reported symptoms during intake, I assessed Adah for depression, PTSD related to physical and sexual assault by her partner, obsessive–compulsive spectrum disorders, ADHD, and perception of reality. Relevant SCID-5 modules were administered. Adah met DSM-5 criteria for persistent depressive disorder related to her visual impairment, unemployment, ostracizing by her siblings, and grief over her mother's passing. She also had PTSD as a result of her persistent partner abuse, generalized anxiety disorder manifested in constant worry about her social status, health, and financial security, and OCD related to fears of contamination. Adah also met the diagnostic criteria for excoriation disorder (skin picking), with visible facial and arm scabs and scars. Furthermore, the SCID-5 indicated a diagnosis of ADHD-Inattentive type. However, schizophrenia spectrum disorders were ruled out based on her SCID-5 findings.

Given Adah's significant mental health challenges, it is highly likely that her motivations to create a compensatory imaginary world were rooted in a deep need for value, security, and care that was lacking in her real-life circumstances. Faced with overwhelming distress from her visual impairment, unemployment, strained family relationships, grief, and partner abuse, Adah may have retreated into an imaginary realm to alleviate her psychological suffering. The diagnoses suggest she was struggling to manage intense emotional turmoil and vulnerability, and the imaginary storylines described below may have provided temporary respite and a sense of control.

### 2.2. The Confabulated Memories

The following three incidents, titled “The new job,” “The miscarriage,” and “The ICU,” are Adah's fabricated memories that were later disproven by her family.

#### 2.2.1. The New Job

In response to persistent encouragement from her father to seek gainable employment, Adah started reporting about meetings online with her career counselor, subsequent job interviews, and eventual hiring for a child-care assistant job. Much to her father's delight, Adah provided detailed descriptions of her experiences as a teacher's aide and shared her enjoyable online interactions with the children. When her father began doubting some of these accounts, he called up the persons allegedly involved, only to find out that none of the events ever happened.

#### 2.2.2. The Miscarriage

Adah called her father one morning during the lockdown period of the COVID-19 pandemic to say that she was bleeding and in pain. She added that she had posted earlier to an online women's support group and written about her condition. Members of the community recommended she talk to a gynecologist. She picked one from the phonebook, and a friend drove her there. A vaginal ultrasound showed two amniotic sacs, suggesting she was aborting twins. She told her father, sobbingly, that the doctor had repositioned the IUD, which was wrongly placed, and that the procedure was painful. Adah then shared her ordeal with her housemate, who was more skeptical and unaware of the nightly drama. After checking the security camera footage, her housemate revealed that no one had left the house that night. Bewildered, Adah called the woman she thought had taken her to the hospital for corroboration, only to find out that nothing of the sort had happened.

#### 2.2.3. The ICU

One of Adah's most profound fears was being contaminated by the COVID-19. What she dreaded most appeared to have happened. One night, she called her father in tears to share that she tested positive for COVID-19 and was hospitalized at the ICU because of breathing difficulties and a high fever. Adah's panic and bawling made it difficult for her father to understand her condition's details and exact whereabouts. Because Adah would not hand over the phone to any of the attending staff, her father suspected that his distraught daughter may have again confabulated an anxiety-laden daydream with reality. He called her housemate and asked her to knock on Adah's door. Adah was lying on her bed sobbing and shaking. “…it felt so real. I could swear it happened,” she muttered. “Oh my God, I was sure I was going to die … I am so humiliated.”

Adah expressed deep distress by her confusion of reality with daydreaming. She realized that in her anxiety, she would imagine plausible developments to the point where she was completely immersed in her fantasy, believing it to be true. She stated that she felt disgraced and “panicky” when learning these were misremembered or false memories.

Despite these grave incidents, there was no evidence Adah was suffering from psychotic or substance-related hallucinations or paranormal experiences. Except for migraines resulting in dizziness, there were no neurological symptoms. Adah was not taking psychoactive medication. The impressions of three professionals she consulted with, a psychiatrist, a neuropsychologist, and a clinical psychologist (the author), were that although the patient was not malingering, she presented with apparent features of a factitious disorder. The possibility of malingering was also considered a differential diagnosis because all three confabulations were expected to result in a sympathetic reaction from her father and siblings. However, several features of her clinical presentation convinced the assessing clinicians to rule out the diagnosis of malingering or pseudologia fantastica: the weeping, anxiety, and overall emotional distress she expressed when reporting the miscarriage and the ICU events appeared genuine; she was known in her family as a naïve and honest woman with no history of lying; her bewildered reaction to the refuting evidence and her strong motivation to seek help for her lifelong daydreaming gone awry suggested the refuted fantasies were ego-dystonic.

The woman I met was anxious and ashamed about misattributing her fantasy to real life. She expressed a desperate need to control these embarrassing memory errors. Given her psychological makeup, I concluded that the most appropriate approach to helping Adah involved three treatment arms applied in a directive and concrete intervention style: (1) supportive reassurance that I believed she was not a “pathological liar” as her siblings had accused her; (2) psychoeducation about MD for the entire family and anchoring Adah's MD in the context of visual and social deprivation in conjunction with her multiple sources of mental distress and; (3) an exploration of her errors in memory source attribution and providing her with tips for better differentiation of reality from anxiety-based daydreaming, for example, improving her sense of agency over her daydreaming and her improving her mindfulness about the entry and exit processes from these self-absorptive states, coupled with fact-checking before sharing significant experiences with her family.

Adah could not tolerate her shameful fictitious memories and her misattribution of anxiety-laden fantasies to reality. Once conscious of her confabulations, they became instantaneously ego-dystonic. Consequently, she fully complied with my suggestions and interventions. Second, Adah could not bear the familial criticism and fraternal ostracizing these false alarms had brought upon her. She acquired the necessary skills to abort the troublesome mistaken alarms, and we mutually terminated the treatment after 22 weekly sessions that included two family meetings. A year later, I contacted Adah for a follow-up in preparation for this case report. She was holding a part-time job as a nursery teacher's aide. Adah reported that although she continued to spend much of her free time in her innocent, colorful fantasy worlds, she had not suffered another incident of misattributing a fantasized event to reality. Her MDS-16 score significantly improved at the follow-up, decreasing from 68 at intake to 49. While her scores for ID remained elevated, she reported a noteworthy reduction in items measuring maladaptation. With her permission, I contacted her father, who provided additional information corroborating the improvements she described.

## 3. Discussion

This paper described the development of spontaneous false memories in a visually impaired woman with MD who was also diagnosed with PTSD, OCD, excoriation disorder, GAD, persistent depressive disorder, and ADHD, inattentive type. Difficulties with rote verbal memory were also identified, which may explain her compromised learning and academic performance.

The bi-directional relationship between MD and various psychological conditions, particularly depression and anxiety, is a complex and multifaceted phenomenon [15]. For example, individuals with PTSD may use daydreaming as a coping mechanism to escape from distressing memories. At the same time, those with OCD may experience intrusive and repetitive daydreams related to their obsessions. Conversely, the presence of MD can also influence the course of depression and anxiety [3]. MD may serve as a dysfunctional coping strategy for managing negative affect, leading to increased withdrawal from reality and social interactions, which can contribute to the maintenance of depressive and anxious symptoms. Additionally, the immersive and time-consuming nature of MD can interfere with daily functioning, further exacerbating feelings of distress and impairment. Further research addressing the bi-directional relationship between MD and comorbid disorders is warranted to provide a more comprehensive understanding.

Individuals often experience more than one mental health condition simultaneously, and comorbidities, such as those presented by Adah, are common among DSM diagnoses [16]. Furthermore, Adah exhibits symptoms consistent with evidence demonstrating MD's high comorbidity with ADHD—primarily inattentive type, OCD, depression, and anxiety disorders [14].

The understanding of memory in individuals with visual disabilities remains limited. A study by Eardley and Pring [17] illuminated the significance of nonvisual sensory imagery in the memory of congenitally blind individuals. Additionally, Tekcan et al. [18] revealed that blind participants exhibited reduced memory retrieval and reported a heightened prevalence of auditory imagery compared to sighted participants. Despite these insights, the existing literature lacks evidence concerning the recall quality and modality among individuals experiencing progressive visual impairment. The current case study is the first known report on visual autobiographical misremembrance in a person who has a progressive visual disability.

Adah lacked the eloquence to articulate her reasons for recounting the fabricated stories. While her motivations cannot be definitively known, some hypotheses may be drawn from her reported symptoms and experiences. Adah reported a history of MD since childhood that appeared to serve an emotion regulation function. Her declining eyesight may have inadvertently reinforced absorption in fantasy worlds over external reality. During the pandemic, heightened isolation and anxiety seemed to catalyze more intense daydreaming around her concerns regarding unemployment, her father's disapproval, and fears over COVID-19. These unmet needs for care and connection potentially contributed to the development of her false event memories.

Source monitoring refers to the cognitive processes involved in attributing the origins of memories, knowledge, or beliefs. In the case of Adah, the false memories of daydreamed events as real experiences can be explained by a failure in source monitoring. She may have had difficulty distinguishing between internally generated scenarios and externally experienced events, leading to the misattribution of the source of the memory. According to Johnson et al. [19], source monitoring errors can occur when individuals have limited perceptual or sensory information to differentiate between internally generated thoughts and externally derived experiences. With compromised or absent visual input, sightless individuals may rely more heavily on internal cognitive processes, potentially increasing the likelihood of source monitoring errors. Furthermore, research by Schacter [20] highlighted the role of source monitoring in creating false memories, emphasizing that individuals with deficits in source monitoring may be more susceptible to incorporating imagined or daydreamed events into their recollections of actual experiences.

Adah's account suggests her misremembered events were phenomenologically realistic and experienced from a field perspective. However, a systematic comparison of the characteristics of her genuine and false memories would be needed to make definitive claims.

Regardless of the underlying mechanisms, Adah evidenced apparent distress and impairment from her pseudo-memories. Her intact reality testing allowed insight into their false nature once presented with contradictory evidence. While speculative causal inferences may be drawn, the available clinical data do not permit definitive conclusions about motivational origins.

In sum, all three occurrences probably started as a means to generate respect and compassion but sadly ended in further humiliation and familial seclusion. Care should be taken to avoid overinterpreting and overgeneralizing beyond the objective evidence. Nevertheless, this case report offers the first documentation of the false memories in an individual with MD.

False memories are incorrect beliefs about the past that are experienced as memories [21]. The psychological literature referred to this term, particularly in the debate around recovered memories of childhood abuse [22]. However, Adah's imaginary experiences and false memories were related to current events, not past ones. Her misremembrances had three distinct features: their appearance was spontaneous, did not involve any form of external persuasion, and were concurrent with the alleged incidents. In other words, they were more akin to a psychotic reality distortion than mental representations of purported external past events. Yet, a psychotic disorder was ruled out. Adah continued attributing her mental experiences to authentic memories even when the events had supposedly ended. She held on to her belief in their integrity until confronted with irrefutable contradicting evidence. Adah experienced deep shame over her pseudo-memories, was seen by her family as a “pathological liar,” and expressed concerns about her unreliable judgment. However, the shift in her meta-cognition and her shamed realization that her memories were based on daydreamed events attested to her solid reality testing.

The source-monitoring model [19, 23] proposes that fantasized representations can become false memories due to being mistaken for authentic memory representations. The decision concerning whether a mental picture is from an internal source or a real-life experience is called source-monitoring attribution. Such a decision is made based on the characteristics of the memory experience. Source-monitoring attributions are usually correct because internally and externally generated representations are dissimilar. Even so, source attribution errors happen, and as a result, misperceptions and misremembering may occur. These mistakes happened in the presented case report when recollections from absorptive, vivid daydreams were characterized by intense sensory and affective features that typically characterize memories of authentic real-life events. Heaps and Nash [24] found when comparing recollective experience in true and false autobiographical memories that true memories were rated as more emotionally intense, possessing more explicit imagery than false memories, and richer in recollective experience and descriptive detail [25]. Furthermore, imagery in true memories was viewed from the field perspective, whereas imagery in false memories was viewed from the observer perspective [24].

Although I had not systematically compared Adah's true and false memories, her misattributed remembrances, as accounted to me and corroborated by her father, were experienced from the field perspective, loaded with specific details, and rich with intense emotion. Put differently, Adah's MD was so realistic that memories of daydreamed scenarios were phenomenologically indistinguishable from autobiographical memories. Moreover, her externally generated memories had grown fuzzier as her sight deteriorated. Therefore, compared to her real-life memories, her internally generated memories seemed much more natural and compelling because her deteriorating ophthalmic disease could not impact them.

The neurobiological explanation for this phenomenon can be linked to the concept of sensory compensation and memory encoding [26, 27]. Sensory compensation refers to the brain's ability to reorganize and enhance the processing of remaining sensory information when one sensory modality is compromised. In the case of Adah, her deteriorating sight may have led to a heightened reliance on internally generated mental imagery to compensate for the loss of external visual input. This increased reliance on internal imagery may have strengthened the neural circuits associated with memory encoding of internally generated content, making these memories more vivid and compelling than her diminishing external sensory experiences. Research has shown that the brain regions responsible for processing mental imagery and memory, such as the hippocampus and parietal cortex, can exhibit plasticity in response to sensory loss, potentially leading to enhanced encoding and retrieval of internally generated mental content [27, 28].

In short, in several ways, Adah's incorrect memories were distinct from previous case reports of false memories [28]. For example, they did not involve “recovered memories” of repressed abuse. Her false memories were contemporaneous with the alleged events: one daydream-based memory described a positive wish fulfillment, while the other two concerned health-related traumatic events. Adah was not subjected to persuasive manipulation; her daydream-based memories were spontaneously generated.

## 4. Conclusion

In a study by Rassin et al. [29], respondents who scored higher on fantasy proneness and dissociation measures said they could not distinguish between dream and reality. This paper presents the first reported case of false memories occurring in an individual with MD, a condition associated with fantasy proneness [11] and dissociation [5]. The findings from this single-case report suggest fantasies in MD could potentially be mistaken for actual autobiographical events in some cases. However, given the limitations of a single case study design with a visually impaired patient, these findings should not be overinterpreted as generalizable to the broader MD community. While provocative, more research is needed with more extensive and diverse MD samples before more decisive conclusions can be made about the prevalence of false memories in this population.

Additionally, this case does not directly address the ongoing debate regarding the origins of dissociative disorders (i.e., sociocognitive or trauma) [17, 30]. Therefore, it would be premature to extend the implications of this report to make inferences about other dissociative conditions without further investigation. Given the acknowledged limitations of this single case study, the following can be tentatively suggested: While Adah's vision produced only restricted and blurry images, her brain's ability to generate clear, realistic images was intact. Her internally generated mental images were much easier to remember than those perceived by her ailing eyes. This is because they resembled visual memories of events recorded in her brain when her eyes were healthy.

One possible explanation of the ability of Adah's visual and memory systems to produce vivid daydreams and misattribute them to actual episodic events is the involvement of the brain's default mode network (DMN), which is associated with internal mentation and self-referential processing. Studies have shown that the DMN is involved in mind-wandering and autobiographical memory retrieval, which are essential components of daydreaming and episodic memory [30]. Since Adah was not congenitally blind, her ability to retrieve visual images was probably intact. Thus, her DMN could have played a role in generating vivid daydreams and their misattribution to actual episodic events.

Additionally, the prefrontal cortex, particularly the ventromedial prefrontal cortex (vmPFC), may be implicated in integrating sensory information and memory, potentially contributing to the misattribution of internally generated experiences to actual events. Research has demonstrated the involvement of the vmPFC in self-referential processing and the construction of mental simulations, which are fundamental to both daydreaming and episodic memory [31].

Furthermore, the connectivity between the DMN and other brain networks, such as the salience network and the limbic system, could underlie the aberrant integration of internal experiences with external reality in visually impaired individuals with vivid daydreams. Altered functional connectivity patterns between these networks have been observed in conditions associated with disrupted self-awareness and reality monitoring. These are relevant to the misattribution of internal experiences to external events [30].

In summary, the compensatory mechanism leading to vivid daydreams and their misattribution to actual episodic events in visually impaired patients may involve the interplay of the DMN, the prefrontal cortex, and their functional connections with other brain networks. Further research utilizing neuroimaging and neuropsychological approaches is needed to elucidate the specific neural underpinnings of this phenomenon.

Although the specific brain mechanisms underlying memory source misattribution remain unclear, as a clinician specializing in trauma and dissociation, I was startled by the encounter with objectively verified false memories in one of my patients, Adah. This case motivated me to undertake a more rigorous empirical investigation to assess the prevalence of memories based on MD mistaken for real-life events, particularly among individuals without visual impairment.

## 5. Implications for Policy, Research, and Practice

The presented case study has several implications worth considering. The paper highlights the diagnosis of MD and other psychiatric comorbidities in a visually impaired woman. This suggests that MD can occur in conjunction with different conditions and emphasizes the importance of a comprehensive assessment to address multiple factors influencing the patient's well-being. The optimal treatment approach should involve addressing both the MD and any underlying psychiatric comorbidities that the individual may be self-medicating for with compensatory fantasy.

The patient's shame and distress resulting from her family refuting her recollection of events illustrate the emotional impact of misremembrance and the potential for interpersonal difficulties. Clinicians should address these emotional experiences, working to validate the patient's feelings and help MD patients develop coping strategies to minimize source attributions and navigate these challenging emotions. The case study implies that the patient's deteriorating eyesight may have contributed to her reliance on vivid, internally generated mental images as a way to compensate for her distressful visual limitations. Understanding the relationship between sensory impairments and the development of elaborate fantasies can inform therapeutic interventions to help patients develop alternative coping mechanisms and enhance their engagement with external reality.

While this single case study suggests investigating source monitoring deficits and memory confabulations in MD may have value, substantial research using larger and more diverse samples is needed before solid conclusions can be made. Understanding the specific challenges faced by individuals with MD, especially those with visual impairments, in differentiating between internally generated daydreams and actual experiences is crucial. Therefore, future research should continue to address the complexities of false memories among individuals with MD, taking into account various factors such as emotional processing and sensory impairments. Should future findings replicate the presence of misremembrances in MD, developing specific therapeutic interventions to improve source monitoring may hold promise for some patients.

## Data Availability

Due to the nature of the paper and the ensuing ethical/legal considerations, supporting data are unavailable.
